# Effect of renal support therapy on 5-year survival in patients discharged from the intensive care unit

**DOI:** 10.1186/s40560-020-00481-0

**Published:** 2020-08-18

**Authors:** Henry Oliveros, Giancarlo Buitrago

**Affiliations:** 1 Department of Clinical Epidemiology and Biostatistics, Pontificia Universidad Javeriana, Bogotá, Colombia; 2 School of Medicine, Universidad de la Sabana, Autonorte de Bogota Km 7, La Caro, Chía, Colombia

**Keywords:** Critical illness, Acute ***renal*** failure, Renal replacement therapy, Survival, Mortality

## Abstract

**Background:**

Between 30 and 70% of patients admitted to the intensive care unit (ICU) have acute kidney injury (AKI), and 10% of these patients will require renal replacement therapy (RRT). A significant number of studies have compared the mortality of patients who require RRT versus those who do not require it, finding an increase in mortality rates in the short and medium term; however, few studies have evaluated the long-term survival in a mixture of patients admitted to the ICU.

**Objective:**

To evaluate the impact of RRT on 5-year survival in patients with AKI admitted to the ICU

**Methods:**

Using administrative databases of insurers of the Colombian health system, a cohort of patients admitted to the ICU between 1 January 2012 and 31 December 2013 was followed until 31 December 2018. ICD-10 diagnoses, procedure codes, and prescribed medications were used to establish the frequencies of the comorbidities included in the Charlson index. Patients were followed for at least 5 years to evaluate survival and establish the adjusted risks by propensity score matching.

**Results:**

Of the 150,230 patients admitted to the ICU, 4366 (2.9%) required RRT in the ICU. Mortality rates for patients with RRT vs no RRT evaluated at ICU discharge, 1 year, and 5 years were 35%, 57.4%, and 67.9% vs 7.4%, 17.6%, and 30.1%, respectively. After propensity score matching, the hazard ratio was calculated for patients who received RRT and those who did not (HR, 2.46; 95% CI 2.37 to 2.56; *p* < 0.001), with a lower difference in years of survival for patients with RRT (mean effect in the treated) of − 1.86 (95% CI − 2.01 to to1.65; *p* < 0.001).

**Conclusions:**

The impact of acute renal failure with the consequent need for RRT in patients admitted to the ICU is reflected in a decrease of approximately one quarter in 5-year survival, regardless of the different comorbidities.

## Introduction

The incidence of acute kidney injury (AKI) in patients admitted to the intensive care unit (ICU) varies between 30 and 70% depending on the definitions used in its classification [1–3]. Approximately 10% of these patients will require renal replacement therapy (RRT), which has a considerable impact on the use of health resources, with costs between $11,016 and $42,077 USD per patient [4, 5]. Mortality also varies widely, ranging from 15 to 60% depending on the associated diseases [6, 7]. Approximately 30% of patients discharged from the ICU that required RRT will remain in the most advanced stage of chronic kidney disease (CKD-5) [7–9], which could be related to a lower long-term survival [10–12]. Different studies have established risk factors at the individual level that determine prognosis. These factors have been first grouped into factors that determine the renal reserve with which a patient enters the ICU, such as age, sex, and comorbidities (hypertension, diabetes) [3, 13, 14]. Second are the conditions for which patients are admitted to the ICU, such as shock states, sepsis, heart failure, and cancer [15–17]. Third, factors to which the patient is exposed during the ICU stay, such as antibiotic therapy, contrast media, vasopressors, or fluid resuscitation, and all these interact to determine the severity of renal injury [18].

Although risk factors have been identified in multiple studies, few have monitored the long-term renal function outcomes in patients discharged from the ICU who presented with AKI requiring dialysis. Determining the individual modifiable factors that could change the course of kidney disease has been the main motivation of studies that have addressed this problem.

Our objective was to determine the 5-year survival of patients admitted to the ICU who required RRT compared to patients who did not require RRT to determine the effect of therapy on survival.

## Methods

### Study type of and data sources

A retrospective cohort study was conducted with administrative data obtained from the per-capita payment unit (UPC) sufficiency database of the Integrated Social Protection Information System (SISPRO) of the Colombian Ministry of Health. This database contains event reports of the services provided by insurance companies (Health Promoter Enterprises [EPS]) of the Colombian health system, which covered 22.5 million Colombians in 2012 (48% of the population). The database is highly standardized and contains the codes of the services provided (CUPS), the date the service was provided, age, sex, insurance company, municipality, ICD-10 code, and cost of care. Information on mortality, date of death, and diagnoses associated with the cause of death was obtained from death certificates.

### Population

The study population consisted of Colombian patients older than 18 years who were first admitted to the ICU between 1 January 2012 and 31 December 2013. Patients were identified by procedure codes related to ICU admission (see Annex 1). Patients who in the previous year were admitted to the ICU or who required renal support therapy in any hemodialysis or peritoneal dialysis modalities were excluded.

### Study variables

The main exposure was the requirement for dialysis therapy, and the main outcome was 5-year survival with administrative cutoff of 31 December 2018. Additionally, mortality was determined at ICU discharge and after 1 year and the continued requirement for renal support after 3 months.

In all patients, the demographic characteristics of age, sex, region of care, and the 16 comorbidities included in the Charlson index, in addition to hypertension, were identified, and last, the condition for which they were admitted to the ICU, such as trauma, heart failure, or sepsis. This information was obtained through the construction of algorithms based on the CUPS procedure codes, ICD-10 diagnoses, and records of administered medications (Annex 1).

### Statistical analysis

First, a descriptive analysis was performed to determine the baseline clinical and demographic characteristics of the groups according to exposure to RRT or not, obtaining the calculation of standardized differences. Second, patients were followed from admission to death for at least 5 years, with administrative cutoff of 31 December 2018. The Kaplan-Meier method was used to obtain the survival curves and to estimate the cumulative mortality at three time points: during ICU stay, between ICU admission and the first year, and between ICU admission and at least 5 years. The hazard ratio (HR) was calculated, both crude and adjusted for age, sex, comorbidities, condition for ICU admission, and region of the country using Cox’s proportional hazards regression. The proportional hazards assumption was verified graphically using log (-log (survival probability)) plots and was found to be appropriate. To reduce possible selection biases due to the lack of randomization and control of confounding variables, matching techniques were performed by determining the propensity index according to the recommendations of Austin [19, 20]. Last, the HRs for the matched groups were recalculated according to the recommendation of Cole and Hernán [21] and confidence intervals using bootstrapping.

The propensity score was obtained from a logistic regression model including the following variables: age, sex, geographical location of the ICU, variables contained in the Charlson index [22], presence of hypertension, condition for ICU admission such as trauma, sepsis or heart failure, and use of contrast media. Subsequently, the matching algorithm was applied, with which the mean effect of RRT was calculated in terms of time to death. The different matching methods were compared to obtain the best balance in the baseline characteristics. The methods used were as follows: nearest-neighbor with ratios of 1:1, 1:5, and 1:10; caliper of 0.05 and 0.001; and Kernel. The nearest neighbor method seeks to match the treated and untreated subjects, trying to make the paired scores as close as possible, while the caliper method establishes a range of closeness based on a measurement in terms of the standard deviation of the propensity, usually a value of 0.05 standard deviations [23, 24]. The criterion used to evaluate the best balance of the baseline characteristics between the groups was to obtain standardized differences less than 0.1 and the lowest value in the index by Rubin and Thomas [25]. Next, the 95% confidence intervals were calculated, and robust standard errors were estimated.

### Ethics approval

Patient records were anonymized, and this study was approved for exemption from informed consent rules by the Ethics Review Board of Pontificia Universidad Javeriana, Bogota, Colombia.

## Results

### Descriptive analysis

**Fig. 1:**
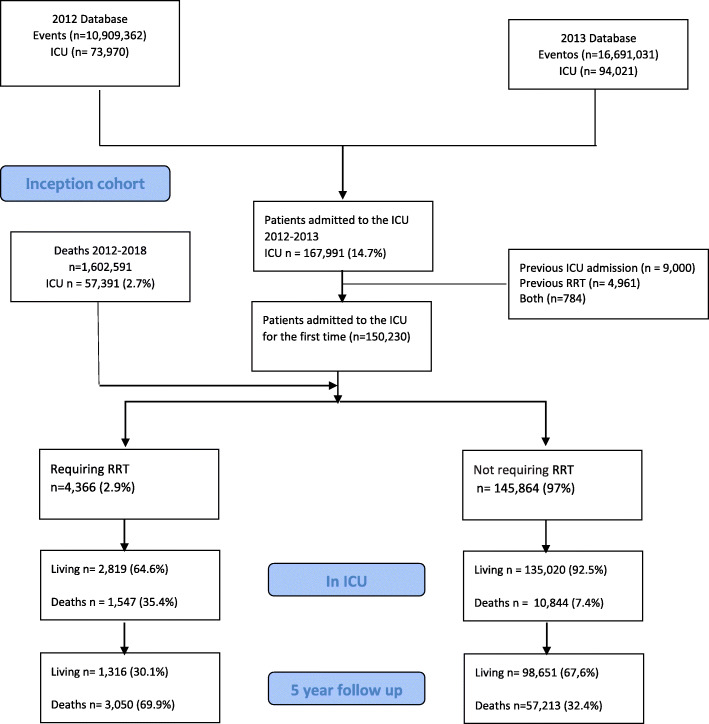
Flowchart of included subjects based on administrative health data

A total of 167,991 patients who were admitted to ICUs in Colombia were identified during the study period, and 17,761 patients with prior admission to the ICU or previous RRT were excluded, with 150,230 patients remaining. Of these, 4366 (2.9%) patients required RRT during ICU stay, as shown in Fig. 1.

The overall mortality at discharge from the ICU was 8.3%, at 1 year was 18.8%, and at 5 years was 31.2%. The two regions that served more than half of the patients were Bogota Central region of the country. Table 1 shows the standardized differences in the baseline characteristics; the group of patients who required RRT had an older age, 67 years (± 15.6) vs 60.5 years (± 12.2), a higher proportion of diabetic patients (33.5% vs 18.7%), and a higher frequency of patients with sepsis (21.6% vs 8.2%). Mortality in patients requiring RRT vs. no RRT evaluated at three time points—at discharge from the ICU, at 1 year, and at 5 years—was 35%, 57.4%, and 67.9% vs. 7.4%, 17.6%, and 30.1%, respectively.

**Table 1:** General characteristics of patients admitted to the ICU from 2011 to 2012 in 180 ICUs in Colombia

Baseline characteristics		Exposure status			
	Full sample	RRT	No RRT	Standardized differences	***P*** value
	***n*** = 150,230	***n*** = 4,366	***n*** = 145,864		
**Age mean (SD)**	60.7 (19.2)	67 (15.6)	60.5 (12.3)	0.373	0.001
Age, *n* (%)					
18–50	39,821 (26.5)	587 (13.4)	39,234 (26.9)	− 0.340	0.001
51–60	22,677 (15.1)	567 (13)	22,110 (15.1)	− 0.062	0.001
61–70	29,727 (19.8)	964 (22.1)	28,763 (19.7)	0.058	0.001
61–70	32,225 (21.5)	1273 (29.2)	30,952 (21.2)	0.184	0.001
71–80	21,951 (14.6)	886 (20.3)	21,065 (14.4)	0.155	0.001
81–90	3784 (2.5)	88 (2.0)	3696 (2.5)	− 0.035	0.031
91–100	45 (0.03)	1 (0.02)	44 (0.03)	− 0.004	0.785
**Region,** ***n*** **(%)**					
Atlantic	19,354 (12.8)	720 (16.4)	18,634 (12.8)	− 0.105	
Bogota	45,615 (30.4)	1,072(24.6)	44,543 (30.5)	− 0.134	0.001
Central	34,241 (22.8)	1060 (24.3)	33,181 (22.8)	0.036	0.018
Eastern	19,789 (13.2)	639 (14.6)	19,150 (13.1)	0.044	0.004
Pacific	30,303 (20.1)	855 (19.6)	29,448 (20.2)	− 0.015	0.326
Other	928 (0.62)	20 (0.46)	908 (0.62)	− 0.022	0.172
Female sex, *n* (%)	75,566 (51)	1786 (40,9)	74,789 (51.2)	0.209	0.001
**Admission to ICU,** ***n*** **(%)**					
Congestive heart failure	10,150 (6.8)	433 (9.9)	9717 (6.7)	0.118	0.001
Trauma	6448 (4,3)	182 (4,2)	6266 (4.3)	− 0.006	0.68
Sepsis	12,854 (8.6)	941 (21.6)	11,913 (8.2)	0.383	0.001
**Comorbidities,** ***n*** **(%)**					
Myocardial infarction	11,983 (8)	276 (6.3)	11,707 (8)	− 0.066	0.001
Congestive heart failure	12,020 (8)	523 (11.9)	11,497 (7.9)	0.137	0.001
Peripheral vascular disease	4977 (3.1)	183 (4.2)	4794 (3.4)	0.048	0.001
Cerebrovascular disease	8043 (5.4)	281 (6.4)	7762 (5.3)	0.047	0.001
Dementia	12,874 (8.6)	334 (7.7)	12,540 (8.6)	− 0.035	0.028
Chronic pulmonary disease	26,218 (17.5)	882 (20.2)	25,336 (17.4)	0.073 hg	0.001
Connective tissue disease	5278 (3.5)	211 (4.8)	5067 (3.5)	0.068	0.001
Peptic ulcer disease	17,101 (11.4)	555 (12.7)	16,546 (11.3)	0.042	0.05
Mild liver disease	1290 (0.9)	68 (1.6)	1222 (0.84)	0.066	0.001
Diabetes mellitus	28801 (19.2)	1463 (33.5)	27,338 (18.7)	0.341	0.001
Metastasis solid tumor	4141 (2.7)	119 (2.7)	4022 (2.8)	− 0.002	0.9
AIDS	682 (0.5)	16 (0.4)	666 (0.5)	− 0.014	0.38
Any tumour	1004 (0.7)	38 (0.9)	966 (0.7)	0.024	0.09
Hypertension	78,898 (52.2)	2904 (66.5)	75,994 (52.1)	0.297	0.001
**Mortality,** ***n*** **(%)**					
Discharge from ICU	12,391 (8.3)	1547 (35)	10,844 (7.4)		0.001
1 year	28,243 (18.8)	2507 (57.4)	25,736 (17.6)		0.001
5 year	46,809 (31.2)	2966 (67.9)	43,843 (30.1)		0.001
ICU stay, mean (SD)	7.1(95)	9.9 (275)	7.0 (96)		0.001
Follow-up time, years	4.5 (2.4)	2.3 (2.7)	4.6 (2.4)		0.001

To calculate *P* values, the chi-squared test was used for categorical variables and Student’s *t* test for continuous variables

The median overall survival time was 2.6 years (95% CI 2.5 to 2.7) for patients requiring RRT and 5.3 years (95% CI 5.22 to 5.25) for patients not requiring RRT.

The characteristics of the groups of patients requiring RRT and not requiring RRT after propensity score matching are presented in Table 2.

**Table 2:** Baseline characteristics in each of the groups after matching

Variable	RRT ***n*** = 4366	No RRT ***n*** = 145,864	Standardized differences	***P*** value
**Age, (%)**				
51–60	12.9	12.6	1.0	0.631
61–70	22.1	22.1	0.1	0.979
61–70	29.2	29.2	− 0.1	0.962
71–80	20.3	21.0	− 1.9	0.398
81–90	2.0	2.2	− 0.9	0.653
91–100	0.02	0.05	− 1.4	0.564
**Region, (%)**				
Bogota	24.5	24.2	0.8	0.709
Central	24.3	24.7	− 0.9	0.672
Eastern	14.6	14.5	0.4	0.856
Pacific	19.6	19.9	− 1.0	0.648
Other	0.46	0.32	1.9	0.303
Female sex, (%)	59.1	58.8	0.6	0.761
**Admission to ICU** ***n*** **(%)**				
Congestive heart failure	9.9	9.9	− 0.1	0.971
Trauma	4.2	4.2	0.0	1.000
Sepsis	21.5	21.7	− 0.3	0.897
**Comorbidities, (%)**				
Myocardial infarction	6.3	5.6	2.9	0.135
Congestive heart failure	11.9	11.7	0.9	0.691
Peripheral vascular disease	4.2	3.9	1.7	0.446
Cerebrovascular disease	6.4	5.5	4.1	0.058
Dementia	7.7	7.8	− 0.4	0.841
Chronic pulmonary disease	20.2	19.5	1.9	0.391
Connective tissue disease	4.8	4.9	− 0.8	0.729
Peptic ulcer disease	12.7	11.6	3.5	0.109
Mild liver disease	1.6	1.2	2.9	0.202
Diabetes mellitus	33.5	32.8	1.6	0.481
Metastasis solid tumor	2.7	2.5	1.4	0.502
AIDS	0.36	0.23	2.1	0.239
Any tumor	0.87	0.96	− 1.1	0.653
Hypertension	66.5	65.6	1.9	0.354
Renal disease	5.7	5.4	1.5	0.574

### Impact of RRT on long-term survival

After matching using different algorithms as shown in Table 3, and after evaluating the common support zone (Figure 1S), the differences in the mean effect between patients who received RRT and those who did not receive it were obtained. The best balance of the baseline characteristics was obtained by the caliper method with a maximum distance of 0.01 SD (RRT years, − 1.86; 95% CI − 2.01 to − 1.65; *p* < 0.001).

**Table 3:** Differences in mean effect on treated patients in terms of survival years according to the matching algorithm

Matching method	Difference RRT years	95% CI Bootstrap	B*	Treated RRT	Controls no RRT	Mortality HR (95% CI)	Difference 5-year mortality %
Nearest neighbor							
1	− 1.85	(− 2.03 to − 1.64)	9.2	4366	2302	2.27 (2.0 to 2.5)	27.5 (24.9 to 30.2)
5	− 1.78	(− 2.00 to − 1.62)	10.4	4366	10,812	2.3 (2.2 to 2.5)	27.5 (23.9 to 31.2)
10	− 1.76	(− 1.98 to − 1.64)	11.4	4366	20,011	2.3 (2.2 to 2.4)	27.6 (23.7 to 31.5)
Maximum caliper distance							
0.01	− 1.86	(− 2.01 to − 1.65)	6.1	4354	2,301	2.46 (2.37 to 2.56)	24.5 (20.7 to 28.3)
0.05	− 2.15	(− 2.02 to − 1.64)	43.9	4366	2283	2.31 (2.06 to 2.59)	26.3 (22.5 to 30.1)
Kernel	− 2.13	(− 2.01 to − 1.66)	41.7	4366	145,864	2.88 (2.77 to 2.99)	26.2 (22.8 to 29.5)

*if *B* > 25%

**Fig. 2:**
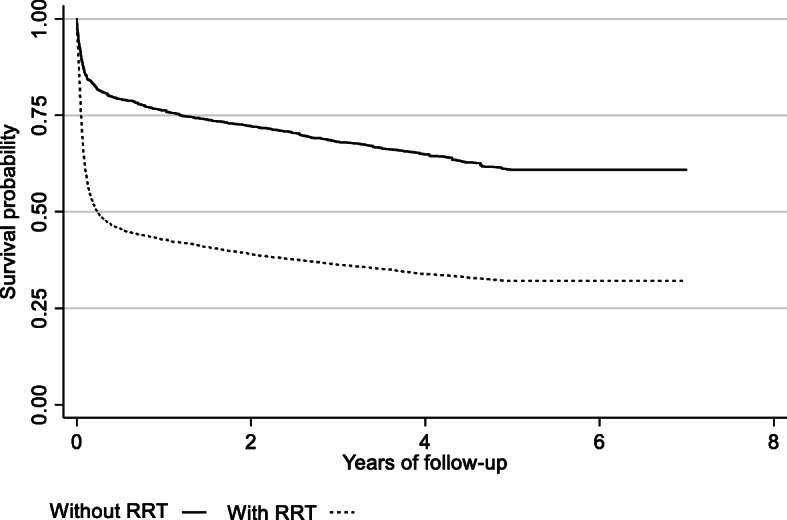
Five-year survival curves of patients requiring RRT and not requiring RRT. Numbers of subjects 150,230, there were 50,263 failures with a rate of 7.4 events per 100 patient-years

Figure 2 shows the unadjusted Kaplan-Meier survival curves of patients requiring RRT and not requiring RRT with a minimum follow-up of 5 years after admission to the ICU. Differences in survival were established early in the first days during hospitalization (Log-rank test, *p* < 0.001).

### Five-year mortality risk of RRT and associated factors

**Fig. 3:**
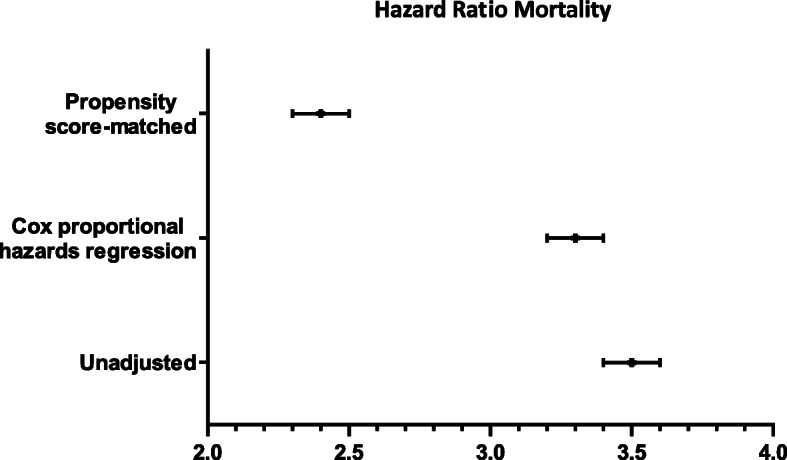
Hazard ratio survival analysis on survival time from ICU admission to death

The 5-year HR for exposure to RRT was calculated, including the crude HR, HR adjusted for the baseline characteristics for the two groups, and HR adjusted by caliper matching of 0.01 SD, respectively: crude HR, 3.40; 95% CI 3.27 to 3.52; *p* < 0.001; adjusted HR, 2.7; 95% CI 2.6 to 2.8; *p* < 0.001; and adjusted HR with matching, 2.46; 95% CI 2.37 to 2.56; *p* < 0.001 (Fig. 3 and Table 3S). The variable that presented the greatest association with mortality was age above 80 years, with an increasing increase for each decade after 50 years (Figure 2S and Table 4S).

*Five-year HR for exposure to RRT: crude, adjusted by the Cox model and adjusted with propensity score matching.*

### Development of CKD and dependence on dialysis therapy

Of the 4366 (2.91%) patients who required RRT, 621 (14.32%) still required RRT by dialysis after 90 days (Table 4 and Figure 3S). The mortality of patients who remained on dialysis vs. those who did not require continuous RRT (CRRT) was 67.88% vs. 32.98%, respectively. However, when the analysis was performed after 90 days and mortality was compared between the subgroup requiring CRRT and patients who did not receive RRT, mortality was 57.33% vs. 32.06%, respectively. After adjusting for confounding by propensity score matching, we compare the mortality in patients that remained with CRRT at 90 days, with patients that did not require RRT due to recovered renal function, we obtained a hazard ratio of 2.4 and a 5-year mortality of 57% vs. 33% for CRRT and RRT respectively.

**Table 4:** Adjusted odds ratio for stay on hemodialysis after 90 days in patients with acute kidney injury

Characteristic	Odds ratio (95% CI)	*P* value
Age, mean (SD)	0.97 (0.96 to 0.98)	0.001
Female sex, *n* (%)	1.40 (1.17 to 1.69)	0.001
Diabetes mellitus, *n* (%)	1.57 (1.30 to 1.90)	0.001
Hypertension, *n* (%)	1.71 (1.37 to 2.15)	0.001
CKD, *n* (%)	1.86 (1.37 to 2.53)	0.001
CPD, *n* (%)	0.62 (0.49 to 0.80)	0.001
Sepsis, *n* (%)	0.46 (0.35 to 0.59)	0.001
Trauma, *n* (%)	0.51 (0.34 to 0.72)	0.016

*P* value corresponds to the association of the independent variable with the permanence in dialysis at 90 days

*CKD* chronic kidney disease, *CPD* chronic pulmonary disease

## Discussion

Using propensity score matching methods, the present study found an HR of 2.46 (95% CI 2.37 to 2.56; *p* < 0.001) with a decrease in 5-year survival of − 1.86 (95% CI − 2.01 to − 1.65; *p* < 0.001) in adult patients who received RRT during their stay in the ICU in Colombia. This result highlights the impact on the Colombian population caused by AKI and its treatment. Several studies have estimated the risk of AKI and RRT in the ICU [9, 12, 18, 26]; however, to our knowledge, there are no studies that have estimated the impact on 5-year survival.

This percentage difference in survival is 24.5% (20.7 to 28.3) lower in patients receiving RRT, which gives an idea of the impact of this condition. There are several studies that do not support this difference. Recently, Abudayyeh et al. [26] studied the impact of RRT in 465 cancer patients admitted to the ICU; mortality at hospital discharge compared with patients who did not receive RRT adjusted by the propensity score showed no differences in the risk of mortality at hospital discharge. Similar findings were observed in patients older than 70 years who required dialysis in the ICU, with no differences in mortality at ICU discharge compared with patients who did not require RRT [27]. While Lebiedz’s study concludes that in 524 critically ill patients with mechanical ventilation requirements, pre-existing CKD has a marked impact on the occurrence of acute renal failure, 30 days and 1-year mortality [28].

The findings of our study are supported on the basis of having obtained large cohort of patients admitted to the ICU that represents approximately 48% of the population served in Colombia in approximately 300 ICUs. Additionally, algorithms were used that covered different dimensions in the identification of the variables. Moreover, in addition to the ICD-10 codes, medical procedure codes and the medications received by the patients were included for the identification of the comorbidities, thus including different dimensions which, as argued by Schneeweiss et al., lead to better control of unobserved confounding variables [29]. The comparison of different matching methods allowed us to choose the algorithm with which the best balance of baseline characteristics between the groups was obtained and thus calculate less biased estimates and with greater accuracy [19, 20, 30, 31]. Nevertheless, we recognize the limitations of not having information on severity scores for the population of patients admitted to the ICU, which would provide us with information about the acute condition of the disease and thus allow us to adjust for severity at the time of ICU admission.

Taking into account a large number of comorbidities and the diagnoses for which the patient were admitted to the ICU, in addition to variables that reflect differences in access and opportunity for health care between the different municipalities of Colombia, allowed us to adjust for a large number of confounding variables [22, 32] and thus obtain less biased estimates, as evidenced in the comparison of the crude and adjusted estimates for the HRs, differences in mean survival times and 5-year mortality percentages (Table 4).

The limitations of the present study are related to the data source, as the data were obtained from administrative databases which ordinarily do not include variables such as biomarkers and severity scores. Some studies that have included biomarkers do not report a discriminatory capacity for the prediction of kidney injury outcomes. Recently, Chen et al. [33] and Malhotra et al. [34] developed predictive models based mainly on clinical characteristics such as sex, age, hypertension, diabetes, coronary heart disease, heart failure, sepsis, mechanical ventilation, total bilirubin, hypoalbuminemia, emergency surgery, cancer, chronic kidney disease, and exposure to nephrotoxic agents, finding an adequate discriminatory capacity (area under the curve of 0.81). In our study, in addition to the comorbidities included in the Charlson index, variables such as diagnosis of sepsis, heart failure or trauma at admission to the ICU, and the use of contrast media during ICU stay were considered.

## Conclusions

The impact of acute renal failure with the consequent requirement for RRT in patients admitted to the ICU is reflected in a decrease of approximately one quarter in 5-year survival, regardless of the different comorbidities. Therefore, attention should be directed towards preventing ICU patients from developing to acute renal failure to improve their prognosis. In the present study, it was observed that dialysis therapy in the ICU alone is not the only factor determining worse outcomes.

## Supplementary information

**Additional file 1: Annex 1.** ICD-10 codes to define Charlson index comorbidities; **Table 3S.** Survival analysis of survival time from ICU admission to death. Mortality hazard ratio ; **Table 4S.** Predictors of mortality by Cox regression model among propensity score-matched patients with RRT compared with those without; **Figure 1S.** Probability distributions according to the propensity score for patients treated with RRT and for patients not treated with RRT logit; **Figure 2S.** Mortality hazard ratio for each variable. CKD: chronical kidney disease; **Figure 3S.** Causal diagrams representing the potential mediation of co-morbidities, ICU interventions, in the association between renal failure requiring dialysis and renal replacement therapy and survival.

## Data Availability

The Information System (SISPRO) of the Colombian Ministry of Health provided the anonymized databases. The refined bases and the code used in the creation of the algorithms were made in STATA MP V.16 and are available to anyone who requests it.

## Abbreviations

AKI: Acute kidney injury
RRT: Renal replacement therapy
CI: Confidence interval
CIAKI: Contrast-induced acute kidney injury
CKD: Chronic kidney disease
CRRT: Chronic renal replacement therapy
ICD-10: International Classification of Diseases, 10th Revision, Clinical Modification
ICU: Intensive care unit
PS: Propensity score
OR: Odds ratio
RR: Risk ratio
SAH: Systemic arterial hypertension
Sdif: Standardized difference
