# Supplementary material for: Effect of renal support therapy on 5-year survival in patients discharged from the intensive care unit
Source: J Intensive Care. 2020 Aug 18;8:63. doi: 10.1186/s40560-020-00481-0 (PMC7437019; doi:10.1186/s40560-020-00481-0)
Supplement: Supplementary file 1 — Additional file 1: Annex 1. ICD-10 codes to define Charlson index comorbidities; Table 3S. Survival analysis of survival time from ICU admission to death. Mortality hazard ratio ; Table 4S. Predictors of mortality by Cox regression model among propensity score-matched patients with RRT compared with those without; Figure 1S. Probability distributions according to the propensity score for patients treated with RRT and for patients not treated with RRT logit; Figure 2S. Mortality hazard ratio for each variable. CKD: chronical kidney disease; Figure 3S. Causal diagrams representing the potential mediation of co-morbidities, ICU interventions, in the association between renal failure requiring dialysis and renal replacement therapy and survival. [file 40560_2020_481_MOESM1_ESM.docx]

**Annex 1. Dimensions taken into account in the construction of the algorithms**

| COMORBIDITY | ICD-10 CODES | CUPS AND MEDICATION |
| --- | --- | --- |
| MYOCARDIAL INFARCTION | I21* I22* I252 I255 | 360100 360101 360102 360102 360200 360201 360202 |
| CONGESTIVE HEART FAILURE | I110 I130 I132 I50* I420 I425 I429 I43* |  |
| PERIPHERAL VASCULAR DISEASE | K551 K558 K559 Z958 Z959 I70* I71* I720 I728 I739 I719 I731 I738 I771 I790 I792 | 380300 380910 383903 392204 392400 392501 392502 392503 392601 392602 392603 392604 392605 380300 392620 |
| STROKE | G45* G46* I60* I69* I67* I681 I682 I688 I694 I698 | 395010 380101 380110 380210 |
| DEMENTIA | F00* F01* F02* FO3* G30* G311 F03X F051 F010 | *RIVASTIGMINE* *DONEPEZIL* *GALANTAMINE* *MEMANTINE* *DONEPEZIL MEMANTINE* *BUPROPION* *AMANTADINE* |
| CHRONIC OBSTRUCTIVE PULMONARY DISEASE | J441 I278 I279 J4* J60* J61* J62* J63* J64* J66* J67* J684 J701 J703 J448 J449 J410 J411 J42X J431 J432 J438 J439 | *OXYGEN* |
| CONNECTIVE TISSUE DISEASE | M053 M058 M059 M060 M061 M063 M069 M050 M052 M051 M353 M320 M321 M328 M329 M353 M330 M331 M332 M339 M340 M341 M342 M348 M349 M353 | *METOTREXATE* *SULFASALAZINE* *LEFLUNOMIDE* *PENICILAMINE* *RITUXIMAB* *ABATACEPT* *ETANERCEPT* *INFLIXIMAB* *ADALIMUMAB* *CERTOLIZUMAB* *GOLIMUMAB* *TOCILIZUMAB* *TOFACIAXIN* *ANAKINRA* *TACROLIMUS* *ACTEMRA* |
| PEPTIC ULCER DISEASE | K270 K271 K272 K273 K274 K275 K276 K279 K250 K251 K252 K253 K254 K255 K256 K257 K259 K260 K261 K262 K263 K264 K265 K266 K267 K269 | S22224 441100 451301 451600 893904 901220 906022 906023 906024 438100 440100 |
| MILD LIVER DISEASE | K709 K702 K703 K717 K740 K742 K746 K740 K742 K746 K743 K744 K745 K730 K731 K738 K739 k70* k73* |  |
| TYPE 2 DIABETES | E100 E101 E106 E108 E109 E110 E111 E116 E118 E119 E120 E121 E126 E128 E29 E130 E131 E136 E138 E139 E140 E141 E146 E148 E149 E140 | *REPAGLINIDE* *NATEGLINIDE* *LIRAGLUTIDE* *EXENATIDE* *LIXIZENATIDE* *DULAGLUTIDE* *ACARBOSE* *MIGLITOL* *CLORPROPAMIDE* *TOLBUTAMIDE* *GLIBENCLAMIDE* *GLIMEPIRIDE* *GLICLAZIDE* *GLIBENS* *GLIPIZIDE* *INSULIN* *INSUMAN* *INSULEX* *INSULIN ASPART* *INSULIN GLULISINE* *INSULIN LISPRO* *INSULIN DETEMIR* *INSULIN GLARGINE* *INSULIN DEGLUDEC* *INSULIN NPH* *METFORMIN* *GEMIGLIPTIN* *EMPAGLIFLOZIN* *PHENFORMIN* *SITAGLIPTIN* *VILDAGLIPTIN* *LINAGLIPTIN* *ALOGLIPTIN* |
| PARAPLEGIA - HEMIPLEGIA | G81 G041 G820 G821 G822 |  |
| CHRONIC KIDNEY DISEASE | I120 I131 N03* N05* Z49* N18 N19 N25 N01 N074 N073 N072  N52 N19 N250 Z940 Z992 | 389500 394300 399501 549002 392701 392702 394200 549001 549012 549800 549801 549802 S22220 S22223 549800 |
| DIABETES MELLITUS WITH COMPLICATIONS | E10* E11* E12* E13* E14* H360 H280 G590 G632 M142 |  |
| ANY TUMOR INCLUDING LEUKEMIA/LYMPHOMA (WITHOUT MALIGNANT SKIN NEOPLASIA) | C000 C001 C002 C003 C004 C005 C006 C007 C008 C009 C01X C020 C021 C022 C023 C024 C029 C030 C031 C039 C040 C041 C049 C050 C051 C052 C059 C060 C061 C062 C069 C07X C080 C081 C089 C090 C091 C099 C101 C102 C103 C104 C883 C887 C889 C900 C901 C91* C92* C93* C94* C95* C96* |  |
| SEVERE LIVER DISEASE | K729 K766 K703 K767 K721 |  |
| METASTATIC SOLID TUMOUR | C780 C7* C8* C781 C782 C783 C784 C785 C786 C787 C788 C79 C790 C791 C792 C793 C794 C795 C796 C797 C798 |  |
| HIV | Z114 Z21X B200 B201 B202 B203 B204 B205 B206 B207 B208 B209 B210 B211 B212 B217 B219 B220 F028 R75X B220 B221 B24* | *ZIDOVUDINE* *LAMIVUDINE* *TENOFOVIR* *EMTRICITABIN* *DIDANOSINE* *NEVIRAPINE* *EFAVIRENZ* *ETRAVIRINE* *SAQUINAVIR* *LOPINAVIR* *ATAZANAVIR* *INDINAVIR* *NELFINAVIR* *RALTEGRAVIR* *ABACAVIR* *ABAMUNE* *RITONAVIR* *ATAZANAVIR* |

**Figure 1S. Probability distributions according to the propensity score for patients treated with RRT and for patients not treated with RRT logit**

**Table 3S. Survival analysis of survival time from ICU admission to death.**

**Mortality hazard ratio**

| **Time period** | **HR Unadjusted* OR (95% CI)** | **HR Adjusted* OR (95% CI)** |
| --- | --- | --- |
| Early mortality in ICU | 1.7 (1.6 to 1.8) | 1.6 (1.5 to 1.7) |
| Late mortality 1 year | 4.3 (4.1 to 4.5) | 3.3 (3.2 to 3.4) |
| Late mortality 5 years | 3.4 (3.3to 3.5) | 2.6 (2.5 to 2.7) |

* Adjusted for age, sex, Charlson comorbidity index, regions of country

**Figure 2S. Mortality hazard ratio for each variable. CKD: chronical kidney disease.**

Figure 3S. Causal diagrams representing the potential mediation of co-morbidities, ICU interventions, in the association between renal failure requiring dialysis and renal replacement therapy and survival

*C2*

C1

C3

ICU

Dialysis

Death Five-year survival

***C1***

*Sepsis*

*Trauma*

*Surgery*

*Medical conditions*

***C2***

*Age*

*Sex*

*Comorbidities*

***C3***

*Fluid balance*

*Hypotension*

*Antibiotics*

*Contrast media*

**Table 4S. Predictors of mortality by Cox regression model among propensity score-matched patients with RRT compared with those without**

| Variable | Hazard Ratio (95% CI) | p value |
| --- | --- | --- |
| RRT: Unadjusted | 3.42 (3.29 to 3.54) | 0.001 |
| RRT: Adjusted by Cox Model | 2.61 (2.51 to 2.71) | 0.001 |
| RRT: Adjusted by propensity score-matched | 2.46 (2.37 to 2.56) | 0.001 |
| **Age** |  |  |
| < 50 | 2.10 (2.01 to 2.19) | 0.001 |
| 50 - 59 | 3.13 (3.01 to 3.25) | 0.001 |
| 60 - 69 | 4.97 (4.78 to 5.16) | 0.001 |
| 70- 79 | 7.61 (7.33 to 7.91) | 0.001 |
| 80- 89 | 10.80 (10.27 to 11.35) | 0.001 |
| 90- 99 | 10.22 (7.29 to 14.33) | 0.001 |
| Female sex | 1.07 (1.05 to 1.09) | 0.001 |
| **Admission to ICU** |  |  |
| Sepsis | 1.04 (1.36 to 1.44) | 0.001 |
| Trauma | 0.96 (0.92 to 1.00) | 0.081 |
| Acute myocardial infarction | 1.11 (1.07 to 1.14) | 0.001 |
| Contrast | 1.89 (1.72 to 2.10) | 0.001 |
| **Comorbidity** |  |  |
| CKD | 1.49 (1.41 to 1.59) | 0.001 |
| Diabetes | 1.16 (1.13 to 1.18) | 0.001 |
| CHF | 1.30 (1.27 to 1.34) | 0.001 |
| Peripheral vascular disease | 1.06 (1.02 to 1.11) | 0.001 |
| Cerebrovascular disease | 1.27 (1.23 to 1.32) | 0.001 |
| Dementia | 1.00 (0.97 to 1.03) | 0.732 |
| COPD | 1.31 (1.29 to 1.34) | 0.001 |
| Connective tissue disease | 1.27 (1.21 to 1.32) | 0.001 |
| Peptic ulcer disease | 1.09 (1.07 to 1.13) | 0.001 |
| Mild liver disease | 1.89 (1.70 to 2.01) | 0.001 |
| Metastasis solid tumour | 2.24 (2.06 to 2.45) | 0.001 |
| AIDS | 1.58 (1.38 to 1.82) | 0.001 |
| Hypertension | 0.92 (0.93 to 0.97) | 0.001 |
| Severe liver disease | 1.32 (1.16 to 1.51) | 0.001 |
| **Region** |  |  |
| Bogota | 0.83 (0.81 to 0.86) | 0.001 |
| Central | 0.94 (0.92 to 0.97) | 0.001 |
| Eastern | 1.07 (1.03 to 1.11) | 0.001 |
| Pacific | 0.96 (0.93 to 0.99) | 0.001 |
| Other | 1.00 (0.87 to 1.13) | 0.95 |

Abbreviations: CI, confidence interval; ICU, intensive care unit; RRT, renal replacement therapy; COPD, chronic obstructive pulmonary disease; CKD, chronic kidney disease; CHF, congestive heart failure
